# Effects of Sevoflurane on the Development of a Human Brain Microphysiological System

**DOI:** 10.3390/ijms27073322

**Published:** 2026-04-07

**Authors:** Qun Li, Lixuan Ding, Itzy E. Morales Pantoja, Navid Modiri, Lena Smirnova, Cyrus David Mintz

**Affiliations:** 1 Department of Anesthesiology and Critical Care Medicine, Johns Hopkins University School of Medicine, Baltimore, MD 21205, USA; imorale4@jhmi.edu (I.E.M.P.); nmodiri1@jhmi.edu (N.M.); 2 Center for Alternatives to Animal Testing, Department of Environmental Health and Engineering, Bloomberg School of Public Health, Johns Hopkins University, Baltimore, MD 21205, USA; lding12@jhmi.edu (L.D.); lena.smirnova@jhu.edu (L.S.)

**Keywords:** anesthesia neurotoxicity, induced pluripotent stem cell, human brain organoid, brain microphysiological system, neural development, mammalian target of rapamycin

## Abstract

Animal studies have shown that early life exposure to general anesthetics may impair brain development. However, the implications of this phenomenon in human patients remain unclear. In this study, we use an induced pluripotent stem cell (iPSC)-derived human brain microphysiological system (bMPS) to investigate the effects of early sevoflurane (SEV) exposure on human brain development. Human iPSCs were cultured and differentiated into neural progenitor cells (NPCs) and then into bMPS. At week 8, bMPSs were exposed to 2.4% SEV for 4 h. Four weeks after exposure, immunofluorescence (IF), Western blotting (WB), and quantitative real-time polymerase chain reaction (qPCR) were conducted to evaluate the alteration of nerve cells in bMPS. After SEV exposure, the number of apoptotic cells increases, and the level of neural differentiation markers decreases. The ratios of mature neurons over NPCs and mature oligodendrocytes over oligodendrocyte progenitor cells (OPCs) are reduced, which leads to a reduction in myelination. SEV also impedes the development of astrocytes and synaptogenesis, especially the formation of excitatory synapses. Meanwhile, SEV increases the expression of molecules in the mammalian target of rapamycin (mTOR) signal pathway. In conclusion, early SEV exposure substantially disrupts the development of human brain tissue, and the mTOR signal pathway is likely to be involved in this alteration.

## 1. Introduction

Over two hundred million general anesthesia procedures are performed each year to allow patients to comfortably tolerate surgeries and procedures that would otherwise be impossible [1], and it is widely accepted that the anesthetic itself has only transient effects on the patient. However, there is concern that some vulnerable categories of patients, especially young children and individuals with underlying brain disorders, might be at risk of lasting neurologic impairments due to toxicity effects caused by exposure to general anesthetic (GA) drugs on vulnerable elements of the brain during critical periods of brain vulnerability [2,3,4]. There is evidence from early human studies in this area that surgery and exposure to GA from late postnatal through early childhood periods may lead to cognitive impairment as measured by an increased incidence of learning disorders and worsening performance on school assessments [5,6]. More recent work has focused on the possibility that early life surgery and GA exposure may correlate with a variety of adverse behavior outcomes [7,8,9]. Based on these studies, the U.S. Food and Drug Administration issued a warning that lengthy or repeated exposure to general anesthetics and sedative drugs from the third trimester of prenatal development through the first 3 years of life may cause disruptions of brain development [10]. Human studies invariably have substantial and unavoidable confounds, including surgery and co-morbid disease, and thus, much of the relevant literature that focuses specifically on the contribution of anesthetics has been conducted in animal models.

**Figure 1:**
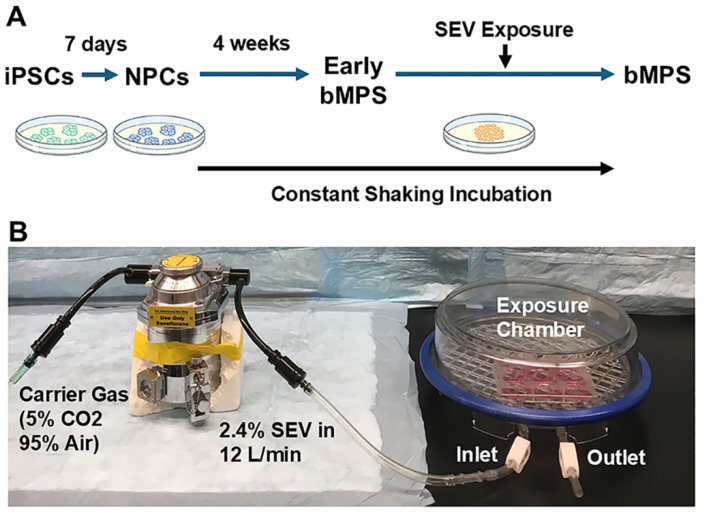
Experimental model system (**A**). Timeline for human induced pluripotent stem cell (iPSC)-derived brain microphysiological system (bMPS) generation. The iPSCs were induced into neural progenitor cells (NPCs) in a 7-day neural induction. NPCs were cultured in uncoated 6-well plates under gyratory shaking (88 rpm) for eight weeks to produce bMPS spheroids. (**B**). Sevoflurane (SEV) exposure at end of week 8. The bMPSs were placed in an air-tight plastic chamber, which was connected to an anesthetic vaporizer that delivers 2.4% sevoflurane mixed with carrier gas (5% CO_2_ and 95% air) for 15 min equilibration. Cultures exposed to carrier gas alone were used as control (CON). Then, the chamber was placed on a gyratory shaker in culture incubator (5% CO_2_) for 4 h. After sevoflurane exposure, bMPSs were removed from the chamber and continuously cultured until the end of week 12.

In rodent and non-human primate animal studies, there is little question that early life exposure to GA agents substantially interferes with brain development and causes lasting neurocognitive and neurobehavioral sequelae [11,12,13,14,15,16,17,18,19]. The preponderance of this literature, and nearly all of our knowledge of putative mechanisms of developmental anesthetic neurotoxicity, is in rodent models. It has been challenging to ascertain how these findings would be expected to translate to human patients, given the substantial interspecies differences in brain structure and developmental timeline. Because cross-species concordance cannot be assumed for neurodevelopmental processes, especially in the context of human brain development, it is important to determine whether the developmental effects of GA described in animal models are also observed in human-relevant systems. Thus, the major contribution of the present study is not primarily the identification of a new mechanism but rather the evaluation of whether known developmental effects of GA extend to a human multicellular brain model, thereby addressing an important translational gap and strengthening the human relevance of this field. To address this question, we tested the central hypothesis that developmental exposure to GA inhibits neural development in a human brain model. We used a human brain cerebral microphysiological system (bMPS), a versatile and novel model well suited for studying developmental neurotoxicity [20], as arguably, it has a high degree of applicability to patients given the use of human-derived iPSC and what is presently available in the basic science armamentarium [21,22]. The bMPS is a three-dimensional (3-D) spheroid culture system derived from induced human pluripotent stem cells (iPSCs) that reproduce multiple key features of human brain development within a single integrated platform. It contains major human brain cell types and supports the interactions necessary for brain development, including proliferation, apoptosis, differentiation, migration, neurite outgrowth, and neural network formation [23,24,25]. Furthermore, the critical elements of CNS structures and functions, such as neuron-neuron interaction (e.g., spontaneous electric field potentials), neuron-glia connectivity (e.g., myelination), and synaptogenesis, are recapitulated. At week 8, the bMPSs were exposed to sevoflurane (SEV) for 4 h. It is not possible to make an equivalence between human chronological age and bMPS days in vitro, given the vast heterogeneity in developmental timelines of the human brain in vivo. We selected 8 weeks in vitro for SEV exposure because, by this time, the brain microenvironment is established, and large populations of neurons and glia have fully differentiated, while neurogenesis and glial development remain ongoing, and the putatively vulnerable processes of synaptogenesis and myelination are still in early stages [26,27]. After 4 additional weeks of growth, the bMPS were harvested and exanimated in week 12, representing a more mature stage of neural development (Figure 1).

In this study, we focused on SEV, the most widely used primary anesthetic agent in early-life pediatric patients. Using quantitative immunofluorescence (IF) microscopy, Western blotting (WB), and quantitative polymerase chain reaction (qPCR), we assessed neuronal growth and differentiation, oligodendrocyte differentiation, myelination, astrocyte production, and synapse formation. We also examined the involvement of the mammalian target of rapamycin (mTOR) signal pathway in this GA-caused alteration in the human brain.

## 2. Results

### 2.1. Effect of SEV Exposure on Gross Development

**Figure 2:**
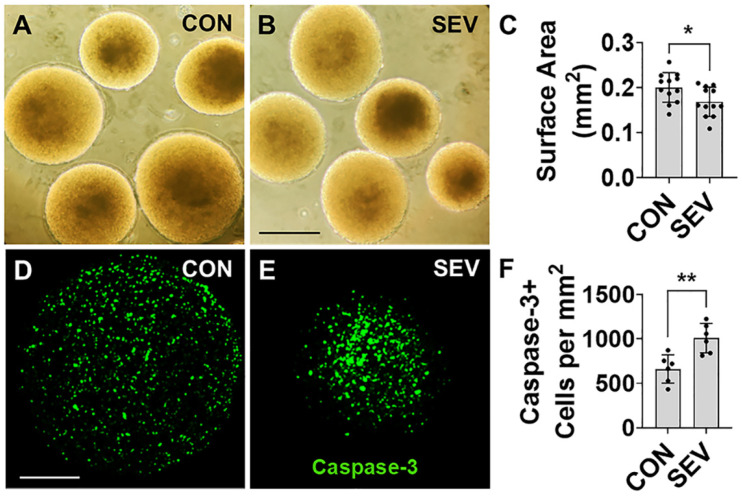
Effect of sevoflurane exposure on bMPS size and apoptosis. (**A**,**B**). At end of week 12 (4 weeks after exposure), bMPSs were observed under light microscope, and photos were taken with 10× magnification. The surface area of bMPSs from control (CON) and sevoflurane exposure (SEV) groups was measured with ImageJ (1.52a). Scale bar = 0.5 mm. (**C**). Quantitative comparison reveals a smaller bMPS size with sevoflurane exposure. Data was obtained from two independent differentiations, each generating 6 bMPSs, for a total of 12 (*n* = 12). Two-tailed Mann–Whitney U test was performed. Dots represent replicates. *: *p* < 0.05. Error bars: SD. (**D**,**E**) The bMPSs were immunostained with cleaved caspase-3 antibody. The apoptotic cells are observed (green). Scale bar = 0.2 mm. (**F**) Quantification of apoptotic cells shown in (**D**,**E**). Data was obtained from 2 independent differentiations, each of which generated 3 bMPSs, for a total of 6 (*n* = 6). SEV significantly increases the number of caspase-3-positive cells. Two-tailed Mann–Whitney U test. Dots in histograms represent replicates. **: *p* < 0.01.

First, we investigated whether SEV exposure affects neural tissue development by measuring bMPS size. To accomplish this, we used light microscopy at the end of week 12 (4 weeks after exposure) to measure the maximal cross-sectional area for each human bMPS and to determine size. In the control (CON) group, the area is measured at 0.20 ± 0.03 mm^2^ (Figure 2A), and it decreases to 0.17 ± 0.03 mm^2^ in the SEV exposure group (Figure 2B). This represents a statistically significant decrease (*n* = 12 from two differentiations, each with 6 bMPSs; *p* < 0.05) (Figure 2C), and it is consistent with a lasting and substantial neurotoxic effect of early developmental anesthetic exposure to human brain tissue.

### 2.2. Effect of SEV Exposure on Developmental Apoptosis

Given the extensive literature suggesting that early developmental anesthetic exposure can cause an increase in apoptosis in a range of different brain cell types [28,29], we counted the cells labeled with cleaved caspase-3, a death protease that is required for the progression of apoptotic processes, in SEV-treated bMPS and CON. A mean density of 662.17 ± 159.03 per mm^2^ of caspase-3-labeled cells is observed in the CON, and this number is significantly increased to 1008.5 ± 164.84 per mm^2^ in the SEV group (*p* < 0.01) (Figure 2D–F). These findings suggest that SEV exposure results in a substantial upregulation of apoptotic pathways in developing human brain cells.

### 2.3. Effect of SEV Exposure on Neurogenesis

**Figure 3:**
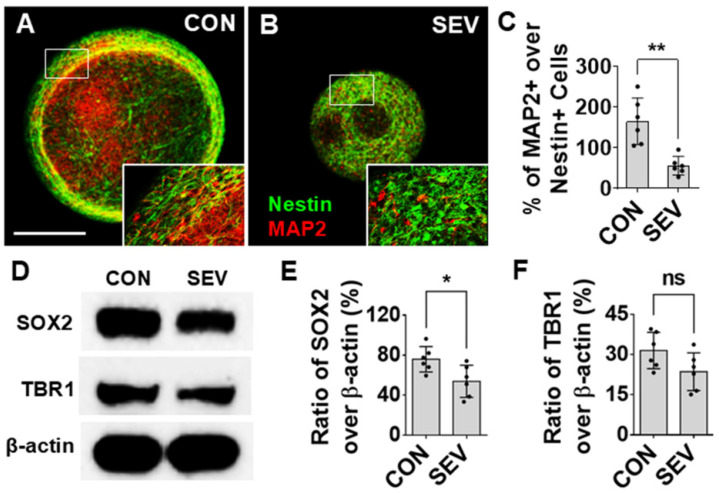
Effects of SEV exposure on neural and neuronal development. (**A**,**B**) Representative images of bMPSs immunostained with Nestin labeled early NPCs (green) and MAP2 labeled mature neurons (red). Boxes in photos (**A**,**B**) represent the locations of high-power images at lower right corners respectively. Scale bar = 0.2 mm. The number of Nestin-labeled cells is increased in the SEV group as compared to the CON group. By contrast, MAP2-positive cell number in SEV is lower than in CON. Consequently, the ratio of MAP2+ cells over Nestin+ NPCs is reduced by SEV exposure. (**C**) Quantitative data for (**A**,**B**); data were obtained from two independent differentiations, each generating 3 bMPSs for a total of 6 (*n* = 6). (**D**). Western blotting images for neural differentiation marker SOX2 and cortical neuronal marker TBR1 in bMPS. SEV decreases the ratio of SOX2 intensity over β-actin, which indicates that neural differentiation is reduced. Reduction in the level of TBR1 means neuronal development is partially impeded. Quantitative results are shown in (**E**,**F**); data were obtained from 2 independent differentiations, each generating three blots (300–450 µg of protein for each) (*n* = 6). Two-tailed Mann–Whitney U tests. Dots in histograms represent replicates. ns: no significance. *: *p* < 0.05; **: *p* < 0.01.

Numerous reports on rodent models using both in vivo and in vitro systems have shown evidence that early exposure to anesthetics can cause alterations in neurogenesis, which, in turn, result in structural changes and functional impairments [30]. The effects of SEV exposure on neurogenesis were evaluated by measuring the density of neural progenitors and mature neurons and by examining their relative proportions as a measure of developmental progression. An IF for the early neural progenitor marker Nestin and the mature neuron marker microtubule-associated protein 2 (MAP2) was conducted (Figure 3A,B). In general, no Nestin+/MAP2+ double-labeled cells were observed, which indicates that Nestin and MAP2 signals represent distinct cell populations, as expected. Overall, the ratio of MAP2+ cells over Nestin+ cells in CON is 164.80 ± 55.87% as compared to 55.82 ± 22.67% in the SEV exposure group (*p* < 0.01) (Figure 3C). Western blotting tests indicate the ratio of immuno-intensity of sex-determining region Y-box 2 (SOX2), the neural differentiation marker, to β-actin in the control (75.92 ± 12.71%), as compared to 53.93 ± 16.06% in the SEV exposure condition (*p* < 0.05) (Figure 3D,E). The intensity of cortical neuronal molecule T-box brain1 (TBR1) in the SEV group is partially lower than that of the control (23.58 ± 7.04% vs. 31.48 ± 6.77%; *p* > 0.05) (Figure 3D,F). While this is not the only possible interpretation, it is suggestive of an impairment in the differentiation of neuronal precursors into mature neurons.

### 2.4. Effect of SEV Exposure on Oligodendrogenesis and Myelin Development

**Figure 4:**
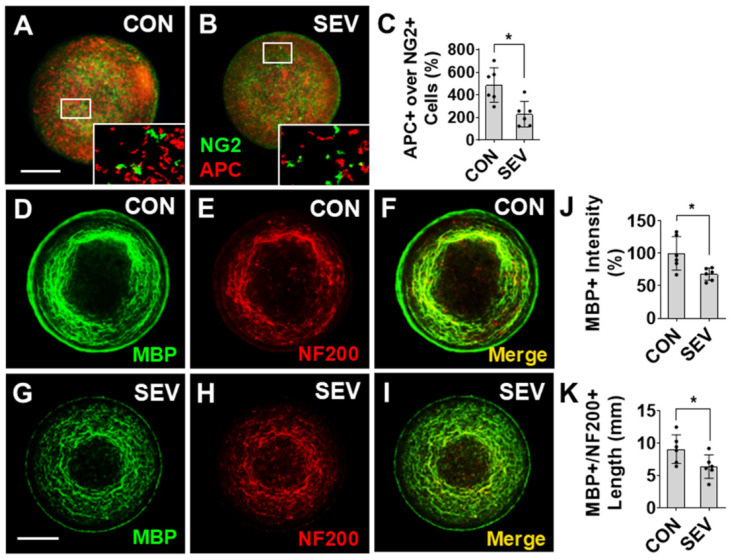
Effect of sevoflurane exposure on oligodendrocyte differentiation and myelination. (**A**,**B**) Representative images of bMPSs labeled with OPC marker NG2 (green) and the mature oligodendrocyte marker APC (red). Insects in (**A**,**B**) show the indicated regions at higher magnification. (**C**) The ratio of mature oligodendrocytes (APC+) to OPCs (NG2+) is significantly decreased in SEV compared with the CON groups, indicating that SEV exposure inhibits oligodendrocyte differentiation. (**D**–**I**) Representative images of bMPSs from the CON group (**D**–**F**) and SEV exposure (**G**–**I**) groups. bMPSs were double-stained with the myelin-specific marker MBP (**D**,**G**) and axonal marker NF200 (**E**,**H**). The intensity of MBP immunoreactivity (**D**,**G**) and the summed length of MBP+/NF200+ double-labeled processes, which represent myelinated axons, were measured in the merged images (**F**,**I**). Compared with the CON group, the SEV group showed a significant reduction in MBP+ immunoreactivity (**J**) and myelinated axon length (**K**). Scale bars = 0.2 mm. Data from all panels were obtained from 2 independent differentiations, each generating 3 bMPSs, for a total of 6 (*n* = 6). Two-tailed Mann–Whitney U tests. Dots in histograms represent replicates. *: *p* < 0.05.

Anesthetic exposure has also been shown to disrupt the formation of myelin through the disruption of oligodendrocyte (OL) differentiation and OL precursor cell (OPC) maturation [15]. To test the effects of anesthetic on OL development, bMPSs were co-immunolabeled with the OPC marker neural/glial antigen 2 (NG2) and the mature OL marker adenomatous polyposis coli (APC) (Figure 4A,B). There are numerous NG2+ and APC+ cells visualized in the bMPSs, but no double-labeled cells are observed, confirming the specificity of the markers. The ratio of APC+ cell number over NG2+ cells in the SEV exposure group (229.85 ± 106.83%) is significantly lower than in the CON conditions (486.75 ± 152.4%) (*p* < 0.05) (Figure 4C). These data suggest that SEV exposure results in a lasting impairment of differentiation of OPCs to the mature OL phenotype.

Next, we investigated the effects of sevoflurane exposure on myelin development using quantitative IF measurement of myelin basic protein (MBP) levels (Figure 4D–I). Compared to CON (100 ± 25.86%), there is a reduced intensity of MBP immunoreactivity in the SEV group (67.82 ± 9.48%; *p* < 0.05) (Figure 4J). Additionally, we measured the sum length of myelinated axons, identified as processes that co-immunolabeled for axon marker neurofilament (NF) 200 and MBP (Figure 4F,I). Early exposure to SEV decreases the total length of NF200+/MBP+ processes (6.39 ± 1.8 mm vs. 9.1 ± 2.2 mm; *p* < 0.05) (Figure 4K). Taken together, these findings suggest that early developmental exposure to sevoflurane results in a lasting reduction in myelination in human bMPS. This is potentially, at least partially, resultant from a reduced number of mature OLs available to provide myelination.

### 2.5. Effect of SEV Exposure on Astrocyte Production

**Figure 5:**
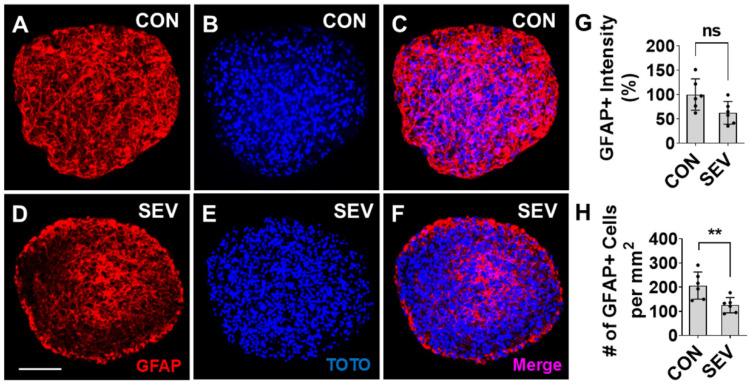
Sevoflurane reduces astrocyte production. (**A**–**F**). Images of bMPSs from CON (**A**–**C**) and SEV exposure groups (**D**–**F**). bMPSs were double stained with GFAP for astrocytes (red) and TOTO for nuclei (blue). Compared to CON, both GFAP reactivity (**G**) and the number of GFAP+ cells (**H**) are reduced in SEV exposure group. Scale bar = 0.2 mm. Data were obtained from 2 independent differentiations, each generating 3 bMPSs for a total of 6 (*n* = 6). Two-tailed Mann–Whitney U tests; Dots in histograms represent replicates. ns: no significance. **: *p* < 0.01.

Previous work in rodent models has shown that astrocytes are also potentially susceptible to the toxic effects of exposure to anesthetics during development [31]. We tested the cell loss of astrocytes by measuring the density of cells positive for the astrocyte marker glial fibrillary acidic protein (GFAP) in our human bMPS model. Compared to CON (Figure 5A–C), a reduction in the density of GFAP+ cells is seen in SEV-exposed bMPSs (Figure 5D–F). We observed that GFAP immunoreactivity partially decreases from 100 arbitrary units ± 32.1% in control conditions to 69.4 arbitrary units ± 23.4% in SEV exposure conditions (*p* > 0.05) (Figure 5G), and the number of GFAP+ astrocytes in the SEV group is lower than in the control (206.5 ± 56.2/mm^2^ vs. 125.3 ± 31.7/mm^2^ *p* < 0.01) (Figure 5H). These findings suggest that early developmental exposure to sevoflurane substantially disrupts astrocyte development in human brain tissue.

### 2.6. Effect of SEV Exposure on Synaptogenesis

**Figure 6:**
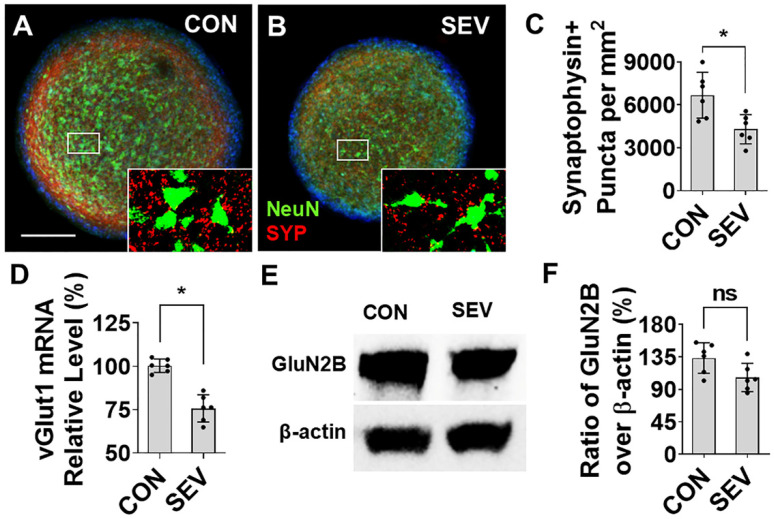
Sevoflurane exposure alters synaptogenesis in human bMPS. (**A**,**B**) Representative images of bMPSs double-labeled for the presynaptic marker synaptophysin (SYP; red) and the neuronal marker NeuN (green). Insets indicate the regions shown at higher magnification. The number of synaptophysin-positive puncta is reduced in the SEV group as compared to the CON group. (**C**) Quantification of synaptophysin-positive puncta. Data were obtained from 2 differentiations, each generating 3 BMPSs, for a total of 6 (*n* = 6). (**D**) qPCR analysis of the excitatory presynaptic molecule vGlut1 mRNA showed that SEV exposure significantly reduced vGlut1 expression compared with control. Data was obtained from 6 differentiations (one well of bMPSs for each, 5–10 mg). Wilcoxon matched pairs signed rank test was performed; *n* = 6, *: *p* < 0.05. (**E**) Western blot analysis shows that SEV exposure reduces the expression of the excitatory postsynaptic protein GluN2B in human bMPS. (**F**) Statistics for (**E**); data were obtained from 2 differentiations, each generating three blots (300–450 µg of protein for each) (*n* = 6). Scale bar = 0.2 mm. Dots in histograms represent replicates. ns: no significance; *: *p* < 0.05.

Numerous in vitro and in vivo animal model studies have shown lasting aberrations in synaptic development, including synapse loss, that result from early anesthetic exposure [13,28]. To investigate the effects of SEV exposure on synaptogenesis, we employed IF to evaluate the intensity of immunolabeled puncta for synaptophysin (SYP), a presynaptic marker, in bMPSs (Figure 6A,B). SEV exposure significantly reduces the number of synaptophysin+ terminals as compared with the control (6679.0/mm^2^ vs. 4298.2/mm^2^; *p* < 0.05) (Figure 6C). Vesicular glutamate transporter-1 (vGlut1) is a representative excitatory presynaptic marker. The mRNA of vGlut1 was examined with quantitative real-time polymerase chain reaction (qPCR). SEV dramatically reduces the relative expression of vGlut1 compared to CON (76.6 ± 8.7% vs. 100 ± 4.1%; *p* < 0.05) (Figure 6D). The Western blotting data shows that the ratio of intensity of glutamate NMDA receptor-2B (GluN2B), a post-synaptic component molecule, over β-actin partially declines in SEV compared with CON (106.5 ± 19.6% vs. 133.3 ± 21.1%; *p* > 0.05) (Figure 6E,F). These findings indicate that early developmental exposure to anesthetics causes a lasting and substantial reduction in presynaptic puncta density and synaptic marker abundance in human brain organoids.

### 2.7. Effect of SEV Exposure on Mammalian Target of Rapamycin (mTOR)

**Figure 7:**
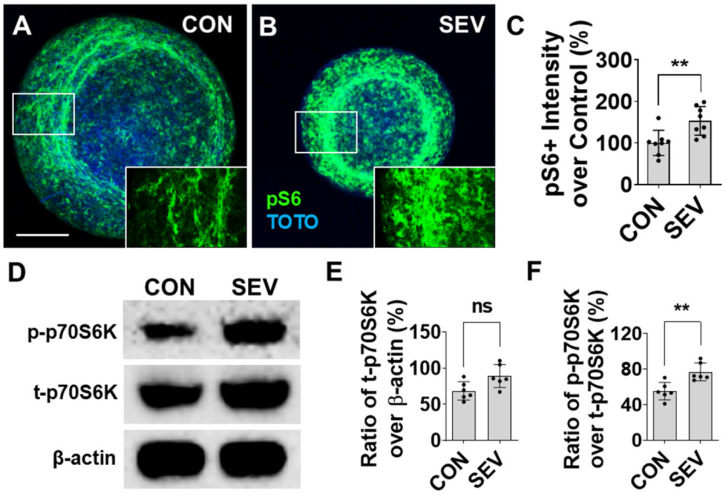
Effect of sevoflurane exposure on mTOR signaling pathway. (**A**,**B**) The bMPSs were immunostained for pS6, a representative marker for mTOR pathway activity. pS6 immunoreactivity was detected in all cellular components, including neurons, glial cells, and axons. (**C**). The intensity of pS6 immunoreactivity of pS6 was evidently higher in the SEV group than in the control group, indicating that sevoflurane exposure aberrantly increases the expression of mTOR pathway. Scale bar = 0.2 mm. Data were obtained from 2 independent differentiations, each generating four bMPSs, for a total of 8 (*n* = 8). (**D**). Western blot analysis shows the expression and phosphorylation of p70S6 kinase, a specific downstream effector molecule. (**E**,**F**) SEV exposure partially enhances the level of total (t-) p70S6K and significantly increases the level of phosphorylated (p-) p70S6K. Data were obtained from 2 independent differentiations, each generating three blots (300–450 µg of protein for each) (*n* = 6). Dots in histograms represent replicates. ns: no significance; **: *p* < 0.01.

The expression of molecules related to the mTOR signaling pathway was evaluated by immunofluorescence (IF) and Western blotting (WB). Our previous data showed that GA exposure pathologically upregulates the expression of the mTOR signal pathway in the rodent model. The phospho-S6 ribosomal protein (pS6), a critical downstream molecule, is used as a marker to evaluate the mTOR level in this study. The pS6 was predominantly distributed in the outer layer of bMPSs and located in both neurons and glial cells (Figure 7A,B). Compared to the control (100 ± 30.13%), the intensity of pS6 reactivity in SEV-exposed bMPSs increases to 153.28 ± 34.58% with a statistical difference (*p* < 0.01) (Figure 7C). WB data show that SEV exposure also alters the expression and phosphorylation of mTOR-related signal molecule ribosomal protein 70 S6 kinase (p70S6K) (Figure 7D). In SEV conditions, the ratio of intensity of total p70S6K (t-p70S6K) over β-actin was partially increased over the CON (89.15 ± 14.67% vs. 68.53 ± 11.53%; *p* > 0.05) (Figure 7E), and the ratio of phosphorylated p70S6K (p-p70S6K) over t-p70S6K in the SEV group is significantly elevated compared with the CON (76.84 ± 9.17% vs. 55.16 ± 8.91%; *p* < 0.01) (Figure 7F). These data indicate that SEV exposure predominantly upregulates phosphorylation of mTOR signal molecules.

## 3. Discussion

In this study, we report that sevoflurane exposure in human induced pluripotent stem cell (iPSC)-derived bMPSs causes a substantial disruption in the normal brain cell development. The human bMPS consists of a variety of cell types found in the human brain, including neurons, oligodendrocytes, and astrocytes; precursor cells associated with these cells - such as neural progenitors and OPCs; cell-type specific specializations, including myelin; and synapses - are critical for neural transmission [24,25,26,27]. This study used a single iPSC line, which limits the assessment of line-to-line variability in developmental trajectory and response to GA exposure. Validation of additional lines will be important to determine the general applicability of the observed effects. Within this human-relevant experimental framework, we next defined the anesthetic exposure conditions to evaluate how sevoflurane affects ongoing neurodevelopmental processes. We chose a 4 h exposure to 2.4% sevoflurane using a previously established in vitro exposure system [28,29,30,31,32]. In our preliminary experiments, we tested a series of sevoflurane doses (1%; 2%; 2.4%; 4%; 8%). The 2.4% sevoflurane concentration chosen in this study is the lowest dose that causes significant developmental alterations in human bMPSs. In addition, this dose corresponds to similar experiments from other researchers, in which the minimum alveolar concentration (MAC) of mice was tested, and a comparison of the water/gas partition coefficient of sevoflurane in vitro and the blood/gas partition coefficient in vivo was performed [33,34]. We did not record the anesthetic concentration stability over time in this study. However, according to our previous research using the same exposure technique, anesthetic concentration stability over time should be ensured [35]. We realize that analysis of anesthetic concentration in the culture medium tested with HPLC will be necessary in our further studies.

Given that the one MAC of sevoflurane varies between 2% and 3.3% during birth and 3 years of life [36], this represents our intent to model the effects of a single, relatively high-dose, lengthy general anesthetic free from the confounds of physiological perturbation. We found evidence of gross perturbation in development, enhanced chronic apoptosis; impaired progression of neuron and oligodendrocyte development; reduced numbers of mature neurons, oligodendrocytes, and astrocytes; and a loss of synapses. While these findings do not prove that anesthetics are toxic to humans in vivo, nor do they speak to the scope of putative toxicity, they do establish that the phenomenon of developmental anesthetic neurotoxicity is not limited to animal model systems and does occur in developing human brain tissue.

Although the predominance of animal studies has been on rats and mice, studies have been conducted, on a limited basis, on non-human primates, and they have shown that general anesthetic exposure in early development causes impaired neurocognitive performance and pathological alteration of the CNS structure [7,8,9,10,11,12,13,14,15,37,38]. However, while animal models recapitulate human brain development in many respects, there are many key differences, both at the cellular and systems levels, including divergent developmental timelines, distinct genetic and epigenetic properties, and substantially dissimilar degrees of structural complexity. To date, conclusive evidence of neuropathology resulting from developmental anesthetic neurotoxicity has not been established in human studies, and it is unlikely that this will occur in the near future, given that it would depend on non-invasive and harmless measurement techniques and would also require rigorous controls that are practically challenging to establish. Recent advances in stem cell culture models, such as the bMPS model we employed, provide the best currently available alternative. In this system, iPSCs simulate the process of human CNS development, including early-stage neural tube formation, neuroepithelium differentiation, and regional specification, in a three-dimensional structure that establishes spontaneous neural transmission and activity. This model has been used to evaluate neurodevelopmental disorders (NDDs) as diverse as microcephaly, autism, and focal epilepsy, as well as other neurological diseases [39,40]. Cultured cerebral organoids from patient-specific iPSCs or gene interference display a variety of microcephaly characteristics, including size reduction, a decrease in neuron number, and enhanced cell death [20,41,42]. In another study of Zika virus-infected organoids, pathological alterations resembling microcephaly, including smaller bMPS size, increased cell death, reduced neural progenitor proliferation, and decreased neuronal cell-layer volume, were observed [43,44,45]. Our data showed similar pathological alterations to other NDDs tested in this model system at a gross level—the size of bMPSs that were exposed to sevoflurane was smaller than that of the controls. We found increased levels of apoptosis; reduced progression of progenitor cells; and ultimately, a loss of mature neurons, oligodendrocytes, and astrocytes, suggesting that these factors account for the observed gross loss of bMPS size. Obviously, this phenomenon has not been observed in humans who were exposed to general anesthesia during brain development—if microcephaly were a consequence of anesthesia exposure, this would surely have been discovered. However, unlike many other NDDs that have been studied with bMPSs, anesthetic exposure is a transient toxic stimulus that presumably alters the developmental trajectory of a cohort of vulnerable cells, rather than a continuously present insult that interferes with most or all cells in the brain, and hence, a subtler phenotype would be expected in vivo.

Although most investigations of developmental anesthetic neurotoxicity have focused on neurons [5,12], brain function and development are heavily dependent on myelin-forming oligodendrocytes, which undergo critical developmental events during the putative window of vulnerability to anesthetic toxicity. Myelination is essential in establishing connectivity in the growing brain by facilitating rapid and synchronized information transfer across the nervous system. Myelin, rather than simply providing insulation to facilitate signal transduction in the developed brain, is critically important for the development of brain circuitry [46]. Myelination involves proliferation and differentiation of oligodendrocyte progenitor cells (OPCs), maturation of oligodendrocytes (OLs), and ensheathment of axons. Damage or loss of myelin function is associated with many neurologic and psychiatric disorders, including multiple sclerosis, Alzheimer’s disease, Parkinson’s disease, and CNS injury [47,48,49,50]. Currently, most of our understanding of neurotoxicity related to oligodendrocyte development and myelination is based on animal studies [15,51], but recent work in brain organoids has begun to extend this work to human tissue [26,52,53,54]. Proliferation and differentiation of OLs and the formation of myelination are known to be susceptible to numerous neurotoxic insults, including alcohol [55,56], cuprizone [57], phenylalanine [58,59], and lead [60,61]. Our work in this area showed that GA exposure in a rodent model resulted in chronic impairments of oligodendrocyte proliferation and differentiation in the fimbria of the hippocampus, which were linked to cognitive dysfunction [15]. The present study raises the prospect that a similar phenomenon of impaired OPC function as a cause of reduced myelin generation may be at work in human tissue. We calculate the ratio of mature OLs over OPCs to evaluate OL differentiation, but this does not represent the OL proliferation. In addition, there are other possibilities, such as selective cell loss, differential marker expression, and alteration of OL survival. The underlying molecular mechanisms have not been fully elucidated; however, the potential for doing so in human OPCs and oligodendrocytes now exists.

The other major functional finding in our study is a possible reduction in synaptic number, and it is the most significant, given the critical role that synapses play in neural computation and transmission. However, to confirm the loss of synapses, further electrical–physiological evidence is necessary. This validation for the synaptic effects was not performed in the present study and should be acknowledged as a limitation, even though the model has been shown to be electrically active in our previous publications. Our analysis does not indicate how much of this may be due to putative neuronal loss versus loss of synapse density per neuron, and we would speculate that both factors are likely at work based on previous work in this field. Numerous studies in animal models have demonstrated synaptic loss because of developmental anesthetic neurotoxicity, and multiple mechanisms have been suggested. To the best of our knowledge, this is the first demonstration of synaptic loss related to early anesthesia exposure in human tissue. While we did not pursue molecular mechanisms, numerous explanations related to cellular function are inherent in our results. It is inherently obvious that neuronal loss, either via apoptosis or via failure of neural progenitor cell progression, has the potential to result in synapse loss. Early work in hippocampal and retinal primary neuron culture made it clear that astrocytes or astrocyte-conditioned media promote synaptic development, and more sophisticated work has since confirmed that astrocytes play a key role in the formation and maintenance of synaptic connections during development [62]. Interestingly, this may, to some extent, be a bidirectional relationship in which astrocytes are dependent on synaptic activity to engender their further growth and development [63]. The mechanisms by which astrocytes and synapses interact include numerous trophic and other signaling factors [64]. While less well established, it is also likely that synapse formation and myelin development are engaged in a mutually supportive bidirectional developmental process through activity-dependent modulation of plasticity [65]. We speculate that the effects that are noted in our bMPS model, in which neurons, oligodendrocytes, astrocytes, myelin, and synapses all show developmental disruption, are interrelated and self-reinforcing.

Our previous work on rodent models has indicated that exposure to general anesthetics (GAs) disrupts neural development in the early postnatal period by inappropriately increasing activity in the mTOR pathway, and both behavioral and histological changes could be reversed by mTOR inhibition [13,15]. In the present study, as in animal studies, we observe a lasting alteration in the tone of mTOR signaling downstream molecules in both neurons and glial cells in bMPS after SEV exposure, which is probably related to developmental disruption. The mTOR pathway is an intracellular signaling pathway that regulates cellular activities, including proliferation, differentiation, apoptosis, metabolism, transmitter release, and other biological processes [66]. In the past decade, many studies have implicated mTOR signaling in neural developmental disorders (NDDs) [67]. We propose that the effect of anesthesia neurotoxicity on neural development in the bMPS model should be further studied. Anesthetic-caused genetic alterations of different cellular components, such as neurons, oligodendrocytes, astrocytes, microglial cells, myelin, and synapses, should be detected. Reagents with potential functions to mitigate impairment of the neurotoxicity, such as mTOR inhibitors, should be identified. We hope further investigations in this field with the bMPS model will be conducted.

In our qPCR study of vGlut1 mRNA, six pairs of samples (*n* = 6) were from six independent cultures. However, in IF and WB experiments, replicates were randomly selected from two batches of bMPS differentiations. We realize this is a statistical weakness. We were unable to exclude technical replicates and pseudo-replication, although experimental conditions between the replicates might be different (such as being cultured in different wells or different gel-running and blotting processes). Our findings are only the beginning of what we hope will be substantial further work on cultured human brain tissue models to elucidate the nature of developmental anesthetic neurotoxicity and the mechanisms behind observed phenomena in human tissue. This model system does have limitations, chief among them that development is driven by genetics and non-patterned activity, rather than by external stimulation. Furthermore, the system we chose represents the human forebrain in terms of cell composition and interactions but lacks the organization of any distinct human brain area that occurs in vivo and thus does not allow for investigation of the development of specific brain circuitry that might be deemed particularly vulnerable to anesthetic toxicity. Although the bMPS does not recapitulate all in vivo determinants, including systemic physiology, vasculature, and sensory-driven patterned activity, this model remains valuable for developmental neurotoxicity studies because it recapitulates major neurodevelopmental processes, including proliferation, apoptosis, differentiation, migration, neurite outgrowth, synaptogenesis, myelination, and neural network formation. In addition, although it lacks regional organization, it supports complex neuron–glia interactions in a multicellular environment in which neurons, astrocytes, and oligodendrocytes arise through coordinated developmental waves. Terminology in the field varies, and the word “brain organoid” is often used to refer generically to any human-stem-cell-derived three-dimensional culture. We have chosen to refer to our system as a “brain microphysiological system” (bMPS), in contrast to approaches that result in the generation of six distinct cerebral cortical lamina or a hippocampal structure with four distinct cornu ammonis subfields, which we would deem an organoid, and this approach could be employed in future work. While stem-cell-based human brain tissue cultures cannot perfectly model the human brain, we believe they hold great promise, particularly for the investigation of genetic and epigenetic mechanisms of anesthetic neurotoxicity that may be unique to human cells. They are also ideally suited for exploring selective vulnerabilities that may arise from human neurodevelopmental genetic diseases that can be modeled by deriving stem cells directly from patients with the disease background. Furthermore, it will be of great value to discern whether previously established mechanisms of anesthetic toxicity in rodent models translate as a whole or in part in culture systems comprising human brain tissue. Our hope is that these approaches can help address the unanswered questions that remain about how and to what degree the human brain is adversely affected by developmental anesthetic exposure.

## 4. Materials and Methods

### 4.1. Generation of bMPS

Human iPSCs NIBSC8 (passage 10–13, female origin) were kindly provided by the National Institute for Biological Standards and Control, NIBSC, UK. The iPSCs were cultured in mTeSR^TM^ Plus medium on vitronectin-coated plates at 37 °C, 5% CO_2,_ and 5% O_2_. After 80–90% confluency, 2 × 10^5^ iPSCs were seeded as small colonies per well in a Matrigel-coated 6-well plate in mTeSR Plus medium supplemented with 10 μM Y-27632. The next day, the medium was changed to neural induction medium (neurobasal medium and 1X neural induction supplement), and the medium was changed every second day. During the induction period, cell morphology was checked under an optical microscope. After seven days of neural induction, iPSCs differentiated into neural progenitor cells (NPCs) and expanded into the Neural Expansion medium (50% Neurobasal, 50% Advanced DMEM/F12 media, 1X Neural induction supplement, and 5 μM Y-27632) for five passages before freezing the NPC stock. Y-27632 was removed the next day after passing. After passage five, NPCs were cultured without Y-27632. Cultures were transferred to normoxia (~20% O_2,_ 37 °C, and 5% CO_2_), and the medium was changed every second day. Passages 6–15 were used to generate bMPSs. NPCs at 90–100% confluence were treated with Gentle Cell Dissociation Reagent (GCDR) for 5 min at RT, after which the reagent was removed. Fresh Neural Expansion medium was added, and the NPCs were detached and counted. A total of 2 × 10^6^ cells per well were plated in uncoated 6-well plates. After 2 days, the Neural Expansion medium was replaced with differentiation medium (B-27^TM^ Plus Neuronal Culture System, 1% Glutamax, 0.01 μg/mL human recombinant GDNF, 0.01 μg/mL human recombinant BDNF, 1% Pen/Strep/Glutamine). Cultures were maintained at 37 °C, 5% CO_2_ and 20% O_2_ under gyratory shaking (88 rpm, 19 mm orbit) for 8 weeks, with medium changes three times per week (Figure 1A). The procedures were adapted from previously established protocols [25,26,27]. In this study, a total of ten independent experiments, defined as ten independent batches of bMPSs, were used across all analyses, including immunofluorescence (IF) staining, Western blotting (WB) and quantitative real-time PCR (qPCR). Four batches were used for IF staining and six for WB and qPCR analysis.

### 4.2. Sevoflurane Exposure

At the end of week 8, sevoflurane exposure in bMPS was performed in a sterile environment. Six-well plates with cultures were removed from the incubator (5% CO_2_, 37 °C), placed in a fume hood, and randomly divided into CON and SEV groups. The plates were placed in identical air-tight, humidified chambers (Billups-Rothenberg, Del Mar, CA, USA), as previously described [28,32]. Before the exposure day, chambers were thoroughly cleaned with 75% ethanol and UV-irradiated for 24 h. After the plates were placed on the mesh of the chamber (lids were placed beside), the chambers were sealed. Both the gas inlet and outlet remained open at this time. The chambers for the SEV group were connected to an agent-specific tabletop portable anesthetic vaporizer (Supera-Vet, Vaporizer Sales and Services Inc., Rockmart, GA, USA) that delivered 2.4% sevoflurane mixed with 5% CO_2_ and 95% air carrier gas. A calibrated flowmeter was used to deliver carrier gas at a flow rate of 12 L/min. Cultures in the CON group were exposed to carrier gas alone. After a 15 min equilibration, the gas inlet and outlet of the chamber were tightly clipped, and the chambers with cultured bMPSs were placed on a gyratory shaker inside the incubator to maintain the temperature at 37 °C (Figure 1B). According to our previous research, anesthetic concentration stability over time in this exposure technique should be ensured [35]. After 4 h of exposure, the chambers were removed from the incubator. The chambers were unsealed, and the sevoflurane mix was released. The cultures were placed back in the incubator for 4 weeks and maintained under gyratory shaking, with medium changed three times per week.

### 4.3. bMPS Size Measurement

At the end of week 12, cultured bMPSs from both the control and sevoflurane groups were observed in a phase-contrast microscope (Nikon, Tokyo, Japan), and photos were taken at 10× magnification. Twelve bMPSs from each group were randomly selected from two independent batches of cultures, six from each, for area (mm^2^) measurement using ImageJ (1.52a) software. Statistics (*t*-test) were performed with the GraphPad Prism 8 software (Figure 2A–C). Two researchers participated in the experiments. The person performing image analysis was blinded to the experimental grouping in all cases.

### 4.4. Immunofluorescence (IF) Staining

After size measurement at the end of week 12, bMPSs were harvested. After twice washing with 0.1 M phosphate buffer saline (PBS), bMPSs were fixed with 4% paraformaldehyde (PFA) in PBS at 4 °C for 60 min. After 3 × 60 min washing with PBS in 1% BSA, bMPSs were transferred into PBS with 0.1% Triton (PBST) in a 24-well plate. The bMPSs were permeabilized in PBST at 4 °C for 30 min and blocked in 300 μL 100% BlockAid per well on a shaker at 4 °C for 60 min. The bMPSs were incubated with the following primary antibodies in 10% BlockAid/Triton for 48 h at 4 °C: (1) Rabbit anti-caspase-3 (1:100; Abcam; Waltham, MA, USA) for apoptosis. (2) Rabbit anti-Nestin for neural progenitor cells (NPCs) (1:250; BioVision, Milpitas, CA, USA) mixed with mouse anti-microtubule-associated protein 2 (MAP2) for mature neurons (1:250; Millipore Sigma, Burlington, MA, USA). (3) Rabbit anti-neural/glial antigen 2 (NG2) for oligodendrocyte progenitor cells—OPCs (1:200; EMD Millipore, Temecula, CA, USA)—mixed with mouse anti-adenomatous polyposis coli-CC1 (APC-CC1) for mature oligodendrocytes (1:300; Millipore). (4) Mouse anti-myelin basic protein (MBP) for myelin (1:500; Santa Cruz Biotechnology, Dallas, TX, USA) mixed with rabbit anti-neurofilament 200 (NF200) for axon (1:100; Sigma-Aldrich, St. Louis, MO, USA). (5) Mouse anti-glial fibrillary acidic protein (GFAP) for astrocyte (1:400; Millipore Sigma; USA). (6) Mouse anti-synaptophysin (SYP) for synapses (1:250; Abcam, USA) mixed with rabbit anti-neuronal nuclei (NeuN) for neurons (1:300; EDM Millipore, USA). (7) Rabbit anti-phospho-S6 (1:1000; Cell Signaling, Danvers, MA, USA). After 3 × 60 min washes with 800 μL of organoid washing buffer (OWB: PBS with 0.5% BSA and 0.1% Triton), bMPSs were incubated in the following secondary antibodies in 10% BlockAid/Triton for 24 h at 4 °C on a shaker: Alexa 488-conjugated goat anti-rabbit IgG (1:300; Invitrogen, Carlsbad, CA, USA) mixed with Cy3-conjugated goat anti-mouse IgG (1:600; Jackson ImmunoResearch Labs, West Grove, PA, USA) or Alexa 488–goat anti-mouse IgG (1:300; Invitrogen) mixed with Cy3-conjugated goat anti-rabbit IgG (1:600; Jackson ImmunoResearch Labs West Grove, PA, USA). After 3 × 60 min washing with OWB and a 60 min wash with 1% BSA in PBS, bMPSs were moved onto glass slides and cover-slipped with Immu-Mount (Epredia, Runcorn, Cheshire, UK) [15,68,69].

### 4.5. Quantitative Analysis of IF Labeling

A total of four independent batches of cultured bMPSs were used for IF staining. The IF-stained bMPSs were observed and imaged at 10× magnification using a Leica 4000 confocal microscope (Leica, Wetzlar, Germany). In each staining, six or eight (pS6) bMPSs with immunoreactivity were randomly selected from two independent batches of cultures (2–4 from each) for further quantitative analysis. Identical photo exposure was set for both groups. Based on the photos taken from these images, data analysis was quantitatively conducted with ImageJ (NIH, 1.52a) [15,68,69].

#### 4.5.1. Cell and Synaptic Puncta Counting

In IF-staining cases for caspase3, MAP2, Nestin, NG2, APC- and GFAP-labeled cells, synaptophysin-positive synaptic puncta were counted. The criteria for caspase-3-positive cells were labeling dead cell nuclei. For other neural cells, immunoreactivity was located in the cytoplasm and proximal processes, and the profiles of cells were identified. For terminal synapses, synaptophysin-positive puncta, either contacting neuronal cytoplasm/proximal dendrites or not, were counted. Labeled cells/synapses were identified with green (Alexa 488) or red (Cy3) channels. Images (10×) were opened and initialized in ImageJ. First, the region of each single bMPS was outlined using the ‘‘Freehand’’ tool. The “Analyze” and “Measure” tools were selected, and the areas (mm^2^) were measured. Then, the “Plugins,” “Analysis,” and ‘‘Cell Counter’’ tools were selected, and each labeled cell inside the bMPSs was clicked, through which each counted cell was marked, preventing the same cell from being counted twice. The number of counted cells in each bMPS was automatically recorded after selecting the “Analyze” and “Measure” tools. The number of labeled cells per mm^2^ was calculated according to the area of bMPSs. We then used the ratio of MAP2-positive cells over Nestin-positive cells, and the ratio of APC+ over NG2+ cells to evaluate the maturation of neuronal and oligodendrocyte lineage cells. For the study of synaptophysin+ puncta, 40 × high-power images from five fields of each bMPS (up, down, left, right, center) were randomly selected. After puncta counting, the average numbers of five fields were calculated.

#### 4.5.2. Measurement of Intensity of Immunoreactivity

The labeling for MBP, GFAP, and pS6 was quantitatively evaluated by measuring the intensity of immunoreactivity. The images were opened with ImageJ, and an outline of bMPSs was drawn with the “Freehand” tool. The “set measurements” was selected from the “Analysis” menu, and “integrated density” was activated. A region outside the bMPSs was selected as the background. The final intensity of MBP, synaptophysin, GFAP, and pS6 equals the measured density minus background.

#### 4.5.3. Measurement of Myelinated Axons

The summed length of MBP+/NF200+ double-labeled axons was measured with ImageJ. In the double-labeling images, the “Freehand line” tool was selected. All MBP+/NF200+-labeled axons (yellow color) in the images were drawn and digitized by clicking “Measure” from “Analyze”. The summed length of these axons (mm) in each bMPS was calculated. For all analysis, two researchers participated in the experiments. The experimenter performing image analysis was blinded to the experimental grouping in all cases.

### 4.6. Western Blotting (WB)

The bMPSs were lysed and homogenized. Samples were run on Bis-Tris Protein Gels for 1 h. The proteins were transferred to nitrocellulose-blotting membranes. Blots were probed with the following antibodies: (1) sex-determining region Y-box 2 (SOX2) for neural differentiation (1:1000; cell signaling, Danvers, MA, USA); (2) cortical neuronal marker T-box brain1 (TBR1) for cortical neurons (1:1000; cell signaling, Danvers, MA, USA); (3) NMDA receptor 2B (GluN2B) for postsynaptic component (1:1000; cell signaling, Danvers, MA, USA); (4) total (t)-p70S6 kinase (1:1000; cell signaling, Danvers, MA, USA) for mTOR signaling pathway; (5) phosphor (p)-p70S6K for phosphorylation of mTOR molecule (1:1000; cell signaling, Danvers, MA, USA); (6) β-actin as a standard marker (1:2000; cell signaling, Danvers, MA, USA). Blots were visualized using the ECL substrate kit. Images were acquired using the ChemiDoc imaging system and quantified with the ImageJ (NIH) software. The ratios of band density of SOX2, TBR1, GluN2B, and t-p70S6K over β-actin and p-p70S6K over t-p70S6K were calculated. Quantitative analysis was performed with the ImageJ program [15,67,68]. Six independent batches of bMPSs were used for the WB study. For each batch, all bMPSs from the CON or SEV group were lysed with lysis buffer, and the supernatant from each group of each batch was collected into 10–15 small tubes (15 µL, 20–30 mg/mL protein for each) for gel running. For each tested molecule, six blots with immunoreactivity from two batches of six batches were selected for further quantitative analysis.

### 4.7. Quantitative Real-Time PCR (qPCR)

The bMPSs were harvested and immediately frozen with dry ice and then stored at −80 °C. The total RNA samples were prepared with “RNeasy Plus Micro Kit” (Qiagen; Frederick, MD, USA). The cDNA synthesis reaction was performed with 1 µg of total RNA plus “qScript cDNA SuperMix” (Quanta Biosciences; Beverly, MA, USA). The specific human vGlut1 primers (forward: GAGCGCAAGTACATCGAGGA; reverse: CTTGCTGATCTCGAAGCCGA) and h-GAPDH primers (forward: CAGAAGACTGTGGATGGCCC; reverse: CCACCTGGTGCTCAGTGTAG) were applied. The qPCR was performed using Bio-Rad “Sso-Advanced Universal SYBR Green Supermix” (Hercules, CA, USA) in the Bio-Rad CFX96 Real-Time System (C1000 Tauch Thermal Cycler; Hercules, CA, USA). The annealing temperature was set at 53 °C, and the number of cycles was fixed to 40. Gene expression was analyzed using the ∆∆CT method with CT as the threshold cycle. The relative levels of target genes, which were normalized to a naive CT value, were reported as 2^−∆∆CT^ [69,70]. Six independent batches of bMPSs were used for the qPCR study. Because a large quantity of bMPS is needed for this study, the tissue for six repeated tests was collected from six batches.

### 4.8. Statistical Analysis

Statistical analyses were performed using the GraphPad Prism 8 software. Sample size was determined based on prior experience with this experimental design. Samples for IF and WB were taken from two independent bMPS cultures, and these for qPCR were from six independent batches. The sample sizes are reported on a per-group basis. Comparisons of IF and WB between the CON and SEV groups were analyzed using the two-tailed Mann-Whitney U test (nonparametric). Statistics for qPCR were performed with the Wilcoxon matched-pairs signed-rank test. Significance was defined as *p* < 0.05. Data are presented as mean ± standard deviation (SD). The sample size (*n*) for IF represents the number of bMPSs analyzed per group and that for WB means the number of blots. The “*n*” in qPCR tests indicates the number of independent differentiations.

## 5. Conclusions

In summary, we conclude that sevoflurane, a frequently used general anesthetic reagent, substantially inhibits the brain development in a human brain microphysiological system (bMPS) and has lasting disruptive effects on the development of neurons, oligodendrocytes, and astrocytes, resulting in a loss of synapses and reduced myelination. The aberrant over-expression of mTOR signal molecules is probably involved in this alteration.

## Data Availability

The datasets generated during the current study are available from the corresponding author upon reasonable request.

## Abbreviations

iPSC	Induced pluripotent stem cell
bMPS	Brain microphysiological system
NPC	Neural progenitor cell
SEV	Sevoflurane
CON	Control
IF	Immunofluorescence
WB	Western blotting
qPCR	Quantitative real-time polymerase chain reaction
MAP2	Microtubule-associated protein 2
SOX2	Sex-determining region Y-box 2
TBR1	T-box brain1
GA	General anesthetic
mTOR	Mammalian target of rapamycin
pS6	Phospho-S6 ribosomal protein
p-p70S6K	Phosphorylated ribosomal protein 70 S6 kinase
t-p70S6K	Total ribosomal protein 70 S6 kinase
GFAP	Glial fibrillary acidic protein
APC	Adenomatous polyposis coli
OL	Oligodendrocyte
NG2	Neural/glial antigen 2
OPC	Oligodendrocyte progenitor cell
MBP	Myelin basic protein
NF	Neurofilament
vGLUt1	Vesicular glutamate transporter-1
GluN2B	Glutamate NMDA receptor-2B
mRNA	Message ribonucleic acid
NDD	Neurodevelopmental disorder
